# Epidemiology of accidents involving venomous animals in the State of Ceará, Brazil (2007-2019)

**DOI:** 10.1590/0037-8682-0511-2020

**Published:** 2021-02-10

**Authors:** Jacqueline Ramos Machado Braga, Marta Maria Caetano de Souza, Iva Maria Lima de Araújo Melo, Luis Eduardo Meira Faria, Roberta Jeane Bezerra Jorge

**Affiliations:** 1 Universidade Federal do Recôncavo da Bahia, Centro de Ciências Agrárias, Ambientais e Biológicas, Laboratório de Répteis e Anfíbios, Cruz das Almas, BA, Brasil.; 2 Secretaria de Saúde do Estado do Ceará, Núcleo de Vigilância Epidemiológica, Coordenadoria de Promoção e Proteção à Saúde, Fortaleza, CE, Brasil.; 3 Universidade Federal do Ceará, Núcleo de Pesquisa e Desenvolvimento de Medicamentos, Laboratório de Farmacologia de Venenos e Toxinas, Fortaleza, CE, Brasil.

**Keywords:** Snakebite, Public health, Venom, Brazil

## Abstract

**INTRODUCTION:**

Envenomation remains a neglected public health problem in most tropical countries. Epidemiological studies on accidents caused by venomous animals are scarce in the Northeast region of Brazil, mainly in the state of Ceará. The present study aimed to describe the epidemiological features of envenomation cases involving venomous animals in the State of Ceará, Northeastern Brazil, from 2007 to 2019.

**METHODS:**

The online Notifiable Diseases Information System was consulted for data on all envenomation cases involving venomous terrestrial animals. Data collected were evaluated for the number of accidents/year, number of accidents/zoological group, antivenom therapy, zone of occurrence, sex, age-group distribution, and deaths.

**RESULTS:**

A total of 54,980 cases were recorded, with the highest incidence being that of scorpion stings (67.2%), predominantly in women (52.4%; odds ratio=3.6; 95% confidence interval=3.5-3.8), equally affecting people aged 10-19 years and 40-59 years (21.4%), in the urban areas (odds ratio=10.3; 95% confidence interval=9.9-10.8), especially in the rainy months. Snakebites (16.7%) had an incidence of 8.1/100,000 inhabitants, but the highest case-fatality rates were observed in bee stings (1.3%) and spider bites (0.5%). Regarding therapeutic variables, a small percentage of people had access to serotherapy (5.3%).

**CONCLUSIONS:**

This study highlights the accidents caused by terrestrial venomous animals as a public health problem that must be monitored in Ceará. Thus, our findings suggest that preventive actions against scorpion and bee stings should be intensified during the months of higher incidence to improve public policies for patient care.

## INTRODUCTION

Due to the process of urban expansion, the shared spaces between humans and venomous animals are increasing, thereby increasing the risk of injuries^1^. Accidents caused by venomous animals occur frequently and represent a serious public health problem in tropical countries. Given this situation, permanent health surveillance actions should be implemented^2^. 

Data from the National Toxic-Pharmacological Information System (*Sistema Nacional de Informação Tóxico-Farmacológica* - SINITOX) reveal that venomous animals are the second-largest agent of human intoxication in Brazil. However, the lack of clinics, laboratories, and qualified technical staff contribute to the still high number of cases^2^ ^,^ ^3^. Accidents caused by venomous animals, considered as a neglected condition in the world, are increasing every year^4^. In 2010, accidents due to venomous animals was included in the compulsory disease notification list (LNC) in Brazil^5^ ^,^ ^6^. Despite the high numbers, the real magnitude of epidemiological data is still inconsistent in Brazil due to the high rate of underreporting and the omission of data while filling out the notification/investigation forms^1^.

Accidents caused by venomous animals have a major impact on health as they compromise work activities, lead to socioeconomic damage, and impair the quality of life due to the possibility of sequelae and temporary or permanent disability. Thus, knowledge of the profile of the injury is essential to institute adequate vigilance in the prevention of cases and, consequently, deaths^7^. Epidemiological studies related to accidents involving venomous terrestrial animals are still very scarce in the state of Ceará. Considering this aspect, we aimed to describe the epidemiological profile of accidents caused by venomous animals that occurred in Ceará between 2007 and 2019.

## METHODS

### Study area

The state of Ceará, located in the Northeast region of Brazil, is composed of 184 municipalities, with a population of 9,132,078 inhabitants in 2019, with approximately 75% living in urban areas^8^. Its territory covers an area of 148,894.7 km² and it is located in the “*Polígono da Seca,*” characterized by reduced rainfall, high temperatures, caatinga vegetation, and generally fine and salty soils^8^.

### Data collection

This was a retrospective study to describe and analyze the epidemiological characteristics of cases of accidents caused by terrestrial venomous animals, using the Notifiable Disease Information System (SINAN) platform for collecting data covering the period from 2007 to 2019, available on the DATASUS website (SUS Department of Informatics, maintained by the Brazilian Ministry of Health) (Ministério da Saúde, 2019). These data were made available for us by *Coodenadoria de Promoção e Proteção à Saúde/COVIG* of the Ceará State Health Department. We used the following variables: sex, age-group distribution, zone of occurrence, month of occurrence, time from bite until assistance, antivenom therapy, deaths, and case evolution. Data on deaths caused by venomous animals were obtained from the Mortality Information System (Sistema de Informações sobre Mortalidade - SIM) using the search for death category ICD-10 (from external causes), grouped with the codes X20 (venomous snakes and lizards), X21 (spiders), X22 (scorpions), and X23 (bees).

### Ethics Statement

Strict ethical and professional aspects were followed, maintained, and respected as established in the Resolution of the National Health Council no. 466 of December 12, 2012, which recommended that research involving only secondary data in the public domain, without nominal identification of the research participants, does not require analysis by a research ethics committee.

### Data analysis

The data were compiled and tabulated to determine the simple frequencies (n) and relative frequencies (%), and the results are presented in the form of contingency tables and graphs made using the Microsoft Excel 2016 program. The incidence, mortality, and case-fatality rates were also calculated. Demographic and population data from the Brazilian Institute of Geography and Statistics (IBGE) were used to calculate the incidence rates in Ceará for every 100,000 inhabitants. Fisher’s test was used, and odds ratios (OR) were calculated to evaluate the association between the demographic variables and the venomous animal types involved in the envenomation^9^.

## RESULTS

**FIGURE 1:**
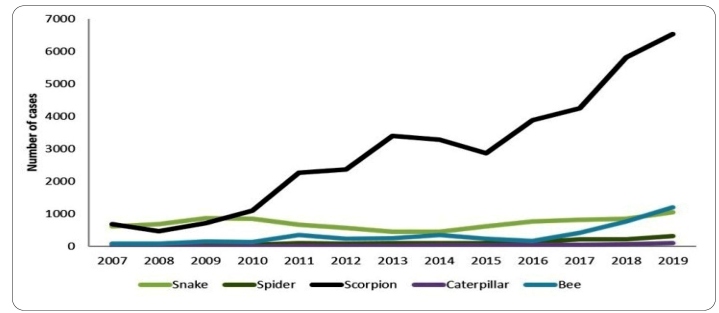
Temporal distribution of accidents involving venomous animals in Ceará, Brazil, from 2007 to 2019*. Source: Sesa/Nuvep/Sinannet, 2020. *Cases in which the venomous animals were not identified were excluded.

In the state of Ceará, there was a marked increase in the number of accidents due to terrestrial venomous animals over the last decade, jumping from 1,492 cases in 2007 to 9,629 cases in 2019^10^. A total of 54,980 cases involving venomous animals were recorded between 2007 and 2019 in Ceará. Of these, 37,606 (68.4%) were caused by scorpions, 9,193 (16.7%) by snakes, 4,375 (8.0%) by bees, 1,551 (2.8%) by spiders, 362 (0.7%) by caterpillars, and 1,272 (2.3%) by other species (Figure 1). 

The state of Ceará recorded the highest number of scorpion stings, ranging from 463 (2008) to 6529 (2019), followed by bee stings, ranging from 84 (2007 and 2008) to 1193 (2019), and snakebites, which ranged from 440 (2014) to 1039 (2019) (Figure 1). 

**FIGURE 2:**
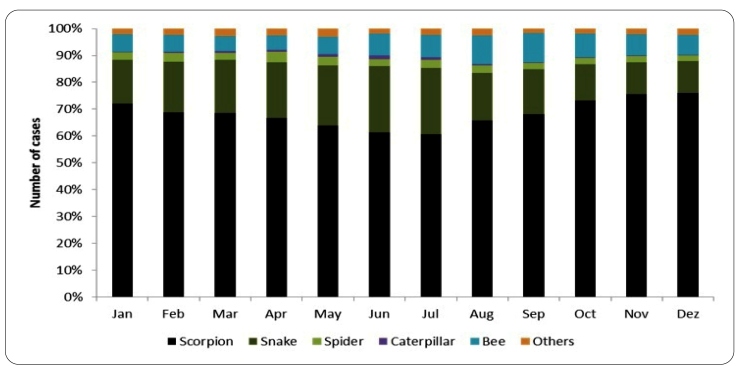
Seasonality of accidents caused by venomous animals, Ceará, Brazil 2007-2019*. Source: Sesa/Nuvep/Sinannet, 2020. *Cases in which the venomous animals were not identified were excluded.

Accidents caused by venomous animals occur throughout the year, following seasonal variations based on the aggressor species. In Ceará, the number of cases of scorpion stings increased from October to January, while envenomation incidents involving caterpillar and snake bites increased between June and July, and bee stings increased between August and September (Figure 2). 

Throughout the historical series in Ceará, the total frequency of cases of envenomation involving women (53.1%) was higher than the frequency of those involving men (46.9%) (Table 1). On analyzing by the type of accident, the most frequently recorded in women were those caused by scorpion stings (81.2%), snakebites (6.9%), and bee stings (5.3%). The same animals also injured men more frequently: scorpions (53.9%), snakes (27.8%), and honeybees (10.9%) (Table 1). An analysis of the association between sex (male and female) and the type of venomous animals showed that there was a higher chance of snakebites in males than in females (OR=5.2; 95% confidence interval [CI] =4.9-5.5; p<0.05). A similar association was revealed with respect to bee stings (OR=2.1; 95% CI =2.0-2.3; p<0.05). As regards scorpion stings, there was a higher chance of it occurring in females than in males (OR=3.6; 95% CI =3.5-3.8; p<0.05).

Table 1 shows the cases distributed by age group, revealing that regardless of animal involvement, the majority of the cases recorded in Ceará occurred in the 29-34-year (35.5%) and 40-59-year (25.6%) age groups (OR=1.2; 95% CI =1.19-1.33; p<0.05). Spider bites (OR=1.2; CI95%= 1.1-1.4; p<0.05) and bee stings (OR=1.9; 95% CI= 1.7-2.0; p<0.05) were higher in the 20-39-year age group. Considering the area of occurrence in Ceará (Table 1) during the study period, the highest frequency of cases was found in the urban area (n=38,373; 71.9%) and was caused by scorpion stings (n=32,075; 83.6%); snakebites accounted for the largest number of cases in the rural area of Ceará (n=7,085; 48.1%). The rural zone showed greater probabilities of snakebites (OR= 1.6; 95% CI = 1.5-1.7; p <0.05), spider bites (OR=1.8; 95% CI = 1.6-2.0; p <0.05), and bee stings (OR=1.7; 95% CI=1.6-1.8; p <0.05), whereas the urban zone showed incidents involving mainly scorpions (OR=10.3; 95% CI = 9.9.10.8; p <0.05).

**TABLE 1:** Sociodemographic data of registered cases of accidents caused by venomous animals in the state of Ceará (2007-2019)*

	Venomous animal								
Variables	Unknown	Snake	Spider	Scorpion	Caterpillar	Bee	Others	Total	
Sex*									
								54,358	100
Male		7,184	729	13,912	165	2,823	660	25,800	46.9
Female		2,008	822	23,694	197	1,552	612	29,175	53.1
**Age range (years)**								n	%
								54,979	100
<1	10	109	29	551	10	66	27	802	1.5
1-4	37	191	62	2048	49	280	86	2,753	5.0
5-9	43	341	65	2044	37	317	96	2,943	5.4
10-14	45	657	83	2123	24	235	86	3,253	5.9
15-19	68	855	164	2984	21	371	98	4,561	8.3
20-39	221	3,101	636	13,017	101	1,995	442	19,513	35.5
40-59	132	2,714	359	9,753	75	791	272	14,096	25.6
60-64	30	434	42	1,576	15	98	43	2,238	4.1
65-69	16	325	32	1,359	11	73	40	1,856	3.4
70-79	14	355	55	1,536	8	99	54	2,121	3.9
≥80	5	107	22	611	11	50	26	832	1.5
Unknown	0	4	1	4	0	0	2	11	0.0
Occurence area*								n	%
								53,332	100
Urban zone		1,801	881	32,075	253	2,586	777	38,373	72.0
Rural zone		7,085	603	4,851	100	1632	445	14,715	27.6
Periurban		55	16	113	9	39	13	244	0.46

*After excluding ignored questions and blank spaces. Source: Sesa/Nuvep/Sinannet, 2020.

Scorpion bites had a higher incidence rate (32.54/100,000 inhabitants), ranging from 5.5 (in 2008) to 71.5 (in 2019). The incidence rate of snakebites was 8.1/100,000 inhabitants, ranging from 5.1 (2013) to 11.4 (2019), followed by bee stings (3.79/100,000 inhabitants), which ranged from 1.0 (2008) to 13.1 (2019). The highest case-fatality rates were observed for bee stings (0.04) and snakebites (0.03). However, the highest mortality occurred in the incidents caused by bee stings (1.9%). For all the venomous animals, the average mortality rate was less than 0.1% and did not show significant changes between 2007 and 2019, regardless of the venomous species involved (Table 2). The majority of victims (n=36,312; 66%) received medical assistance within 3 h after envenomation (Table 2).

**TABLE 2:** Epidemiological data of incidents involving venomous animals (snake, spider, honeybee, scorpion*, and caterpillar**), Ceará-Brazil, 2007 to 2019 (n=54,980).

	Venomous animal								
Variables	Unknown	Snake	Spider	Scorpion	Caterpillar	Bee	Others	Total	
**Time from bite until assistance (h)**								n	%
								54,980	100
Unknown	124	549	160	1,986	30	483	117	3,449	6.3
0-1	121	2,622	325	13,598	123	1,471	346	18,606	33.8
1-3	122	3,311	309	12,473	105	1,106	284	17,710	32.2
3-6	50	1,419	145	4,592	55	341	150	6,752	12.3
6-12	40	639	101	2,686	17	209	89	3,781	6.9
12-24	59	375	183	1,600	17	332	140	2,706	4.9
≥24	105	278	328	671	15	433	146	1,976	3.6
**Antivenom**								n	%
								51,894	100
Yes	105	7,006	248	1,863	8	42	56	9,328	18.0
No	469	2,617	1,201	32,996	328	3,847	1,108	42,566	82.0
**Case evolution**								n	%
								54,980	100
Unknown	135	1334	213	1760	22	316	124	3,940	7.2
Cured	484	7,816	1,326	35,822	340	4,049	1148	50,985	92.7
Death due to the notified condition	0	29	7	2	0	50	0	88	0.2
Death due to another condition	2	4	2	1	0	1	0	10	0.0
**Classification of case**								n	%
								54,980	100
Unknown	157	779	139	1,002	17	270	95	2,459	4.5
Mild	382	5,716	1,103	35,391	323	3,559	1080	47,554	86.5
Moderate	72	2,322	288	1,137	22	512	91	4,444	8.1
Severe	10	376	21	76	0	34	6	523	1.0

Source: Sesa/Nuvep/Sinannet (2020) and SIM (2020) to death records. *There were two deaths from scorpion bites in the historical series, resulting in zero mortality. and case-fatality rates. **There were no reported lethal cases involving caterpillars in the period.

As shown in Table 3, between 2007 and 2019, 54,980 incidents involving terrestrial venomous animals resulted in 88 deaths in Ceará, and the years 2019 (n=14; 15.9%) and 2009 (n=10; 11.4%) showed the highest number of deaths (data not shown). 

**TABLE 3:** Epidemiological variables of registered cases of incidents involving venomous animals in the state of Ceará (2007-2019).

	Snake			Spider			Bee			Scorpion*	Caterpillar*
Year	Incidence	Mortality	Lethality (%)	Incidence	Mortality	Lethality (%)	Incidence	Mortality	Lethality (%)	Incidence	Incidence
2007	7.34	0.01	0.3	0.67	0.00	1.8	1.08	0.05	4.4	8.10	0.04
2008	8.06	0.02	0.3	0.59	0.00	0.0	0.99	0.04	3.6	5.48	0.06
2009	10.12	0.04	0.3	0.68	0.01	0.0	1.77	0.09	5.3	8.41	0.05
2010	9.94	0.05	0.1	0.60	0.00	0.0	1.46	0.02	1.6	13.04	0.02
2011	7.73	0.02	0.2	1.04	0.02	0.0	4.09	0.06	1.4	26.63	0.11
2012	6.50	0.05	0.7	0.91	0.00	0.0	2.66	0.01	0.4	27.38	0.14
2013	5.11	0.02	0.4	1.05	0.00	0.0	2.86	0.02	0.8	38.66	0.24
2014	4.98	0.01	0.7	1.15	0.00	0.0	3.95	0.02	0.6	37.19	0.40
2015	6.86	0.04	0.2	1.12	0.02	0.0	2.52	0.07	2.7	32.10	0.25
2016	8.56	0.03	0.3	1.44	0.03	2.3	1.85	0.03	1.8	43.33	0.44
2017	9.07	0.06	0.2	2.34	0.01	0.9	4.58	0.06	1.2	47.13	0.44
2018	9.35	0.01	0.1	2.39	0.00	0.0	8.35	0.02	0.3	64.05	0.78
2019	11.38	0.08	0.6	3.46	0.01	0.3	13.06	0.08	0.6	71.50	1.08
Average	8.08	0.03	0.3	1.34	0.01	0.4	3.79	0.04	1.9	32.54	0.31

Source: Sesa/Nuvep/Sinannet, 2020. *There were no deaths during the study period.

The highest frequency of deaths was found among the cases caused by bee stings (n=50; 56.8%), snakebites (n=29; 32.9%), and spider bites (n=7; 8%). The case evolution was not identified in 3,940 cases (7.2%). Among all the cases described in the last 12 years, 523 were severe, mainly involving snakes (n=376) and bees (n=34). From 2007 to 2019, antivenom therapy was administered in Ceará in 9,328 (18%) patients, and snakebites (7,006; 75.1%) and scorpion stings (1,863; 20.0%) were the cases that most frequently received antivenom therapy. 

## DISCUSSION

Brazil is one of the countries with the most experience in the diagnosis and treatment of cases involving venomous animals. There are four notification systems for registering envenomations and case-fatality rates in the country. Currently, the SINAN is the most-used tool for analyzing these cases^11^ ^,^ ^12^, but SIM remains the most appropriate system for analyzing mortality data^13^ ^,^ ^14^. 

The SINAN has recorded a progressive increase in the number of notifications involving venomous animals every year in Brazil^4^. Our data reveal an upward trend in the number of incidents involving venomous animals in Ceará. However, it is not possible to analyze whether there has been a real increase in the number of cases over the years or an improvement in the reporting system. This growth trend in cases involving venomous animals is corroborated by studies in other states in the Northeast Region, such as Rio Grande do Norte^15^, Paraíba^2^, and Piauí^16^.

Previous epidemiological studies have confirmed the influence of human and environmental factors on the occurrences of incidents involving venomous animals in Northeast Brazil^17^, especially in the rainiest months^15^ ^,^ ^16^ ^,^ ^18^, corroborating our data. In our study, women were more vulnerable, and these results are in line with those of other epidemiological studies involving incidents caused by venomous animals in Brazil^19^ and in the Northeast region^15^ ^,^ ^17^ ^,^ ^20^ ^-^ ^22^. The predominance of men usually reveals their greater exposure to areas where there are risks of snakebites, and this is especially true for those performing manual civilian work, such as agricultural workers^15^. However, women and children are more exposed to scorpions, spiders, and caterpillars in the residential environment^23^.

Disorganized urban growth and industrialization, irrational use of natural resources, and ecological imbalance predispose the proliferation of venomous animals and promote overlap between the spaces used by humans and animals^24^. The increase in the population in Ceará probably impacted the occurrence of snakebites and scorpion stings, considering the increase in the volume of domestic waste^15^. 

Previous studies in Ceará revealed a high incidence of scorpion bites that may have resulted from the high population density in the capital and from climatic conditions (high temperatures) and urban conditions (accumulation of waste and inadequate sanitation) that would facilitate the adaptation of scorpions to life in urban areas^20^. Thus, the implementation of educational programs for the prevention and treatment of envenomation by scorpions, offered to community and health agents, can be an effective measure of public policy to reduce the growing number of cases.

Mortality due to envenomation by venomous animals is due to the toxicity of the venom, the amount inoculated, and the precocity and effectiveness of the treatment administered to the victim^25^. Children, adolescents, and the elderly are more vulnerable to snakebites and scorpion stings, with higher case-fatality rates^19^. In previous studies conducted in Ceará, it was found that the majority of incidents involving venomous animals occurred in an urban areas^15^ ^,^ ^20^. Our data reveal that bee stings changed the epidemiological picture of Ceará, which shows the importance of studies with this venom and that of updating the notification data so that public policies can be directed to the injured. In previously sensitized people, a single sting of a hymenopteran insect, such as a honeybee, can result in rapid death^26^. However, even in non-atopic people, multiple stings can lead to death due to the higher dose of injected venom^27^ ^,^ ^28^. 

In case of incidents involving venomous animals that occur in the rural areas of many tropical countries in Africa, Asia, New Guinea, and South America, there are difficulties in accessing conventional medical treatment in hospitals, due to delays in transporting the victim or because of the preference for treatment by local traditional healers^28^. Hospitalization occurs in moderate and critical cases that require specialized care due to their prolonged symptoms and the possibility of death^29^. An important factor to note is that most incidents involving venomous animals occur in regions that are far from referral hospitals (rural areas), and delayed care can aggravate cases, possibly resulting in systemic sequelae, the need for amputations, and even death of the victim in a few hours^1^. Such situations that result in death outside the health facilities are not entered in the notification records of the Ministry of Health. Thus, in most cases, the numbers do not portray the reality^28^. 

Treatment time is associated with the severity of the envenomation; therefore, victims must have early access to medical care and antivenom therapy, thus resulting in better prognosis^30^ ^,^ ^31^ ^,^ ^32^. Studies carried out in the states of Bahia^30^ and Piauí^33^showed an improvement in the information provided to the population about the urgency of medical assistance in these cases.

A previous study^18^ revealed that a total of 1,307 cases of bee stings were registered by Ceará in the SINAN, with only four deaths and a case-fatality rate of 0.3%, a value well below that found in our study (1.9%) using SIM death records. Such differences in the notifications of deaths may result from discrepancies in the updating of the mandatory notification systems (SIM and SINAN), which is a problem since SINAN identifies a marked increase in the number of deaths due to scorpion stings. The SIM shows a greater increase in the number of deaths due to bee stings, thus making it difficult to construct epidemiological scenarios^34^.

The underreporting of incidents involving venomous animals has been stated by several studies^17^ ^,^ ^35^. However, the increase in the number of cases over the years may reflect an improvement in the reporting system. Although Brazil has four national systems for recording, the information is still dissociated, resulting in data that may not represent the reality of this public health problem^35^. Thus, there is a need to analyze and periodically evaluate these notification systems, considering their strategic importance in decision-making in health service routines, public policies, and research^34^ ^,^ ^36^.

Up-to-date regional information is important for the development of epidemiological surveillance. A better understanding of the epidemiology of incidents caused by venomous animals in the state of Ceará should facilitate their prevention and management. Thus, more detailed studies are needed to clarify the clinico-epidemiological profile of envenomations, which will assist in the implementation of educational health care measures seeking to improve the assistance provided to victims.
